# Well Pattern optimization as a planning process using a novel developed optimization algorithm

**DOI:** 10.1038/s41598-024-78196-7

**Published:** 2024-11-05

**Authors:** Seyed Hayan Zaheri, Mahdi Hosseini, Mohammad Fathinasab

**Affiliations:** 1 National Iranian Oil Company, Ahvaz, Iran; 2 Research Institute of Petroleum Industry, Tehran, Iran

**Keywords:** Genetic algorithm, Particle swarm optimization, Reservoir Simulation, Well Placement Pattern, Chemical engineering, Energy infrastructure, Mechanical engineering

## Abstract

Determination of optimum well location and operational settings for existing and new wells is crucial for maximizing production in field development. These optimum conditions depend on geological and petrophysical factors, fluid flow regimes, and economic variables. However, conducting numerous simulations for various parameters can be time-consuming and costly. Also, due to the high dimension of the possible solutions, there is still no general approach to address this problem. The application of searching algorithm as a general approach to solve such problems has received much attention in recent years. In this study, the efficiency, and reliability of genetic algorithm, particle swarm optimization and in particular a newly developed algorithm was analyzed and compared. The novelty of this work is the integrated algorithm, which improves searching performance by leveraging the memorizing characteristics of the particle swarm optimization algorithm to enhance genetic algorithm efficiency. In traditional genetic algorithms, solutions lacking adequate qualifications are deleted from the algorithmic process; however, the new algorithm provides these solutions with additional opportunities to prove themselves by acquiring new velocities from particle swarm optimization. The results indicate that while the genetic algorithm and particle swarm optimization do not guarantee optimal outcomes, the newly developed algorithm outperforms both methods. This performance was tested across various scenarios focused on well pattern optimization, highlighting its innovative contribution to the field development.

## Introduction

Traditional methods in reservoir fluid production, produce only about 20–30% of the initial oil in place during the primary production stage whereas this value increases to 40% during secondary production^1^. The worldwide energy requirement raises the necessity for the development of methods to maximize oil recovery from the remaining 60% of the original oil in place. Developing a plan that results in maximum recovery from an oil or gas field, considering the physical and economic constraints, is one of the main duties of a reservoir engineer^2^. On the other hand, finding an optimum well location, arrangement, and other operational conditions could help in planning a schedule to maximize net present value, calculated according to the oil price and production profile versus time^3^.

Since there are many parameters and variables considered as inputs into the simulation system in reservoir modeling to provide a production forecast profile, it is possible to obtain different production profiles from different suggested sets of configuration and placement. In order to determine the optimum solution, that is capable of providing the best strategy from the economical point of view, iterative methods can be employed effectively^3^. Using the optimization techniques in well placement goes back to more than two decades ago^2,4–6^. Generally, the previously published works on optimizing well placement and field development can be classified into three main areas; Development of frameworks and algorithms for optimizing well placement^7–10^; Creation of proxies to expedite the optimization procedure^5,11,12^; and evaluation of uncertainty and its integration into the optimization process^13,14^.

During an iterative method, depending on input variables, the optimization problem defines an “objective function” in which the target values are optimized based on the best solution. In the case of determining the best well placement, usually, the objective function is the net present value (NPV)^15,16^or cumulative field production^17^over a defined period, and the input factors are the parameters that describe potential locations, spacing between wells and small displacement around them, infill drilling after a special period, length and direction of horizontal well all in the well pattern planning process^18–20^.

Genetic algorithm is one of the optimization algorithms employed in well placement optimization by numerous researchers^21–24^. The potentiality of GA has made it the predominant optimization algorithm used for well placement and other reservoir-management applications^4,25^. Montes et al^26^. applied standard genetic algorithms (GAs) for enhancing the positioning of vertical wells that traverse the entire vertical domain, with the goal of maximizing total oil field production. Emerick et al^21^. developed a computer-aided optimization tool utilizing a Genetic Algorithm to optimize the number, location, and trajectory of producer and injector wells. Two strategies for defining the initial population were tested: one using random configurations, and another based on an engineer’s proposed case. The latter yielded superior results, effectively enhancing the initial design. Morales et al^27^. proposed a modified genetic algorithm for optimizing well placement under geological uncertainty, enabling users to define acceptable risk levels. The algorithm evaluates multiple geological models to identify the fittest well location based on cumulative production or net present value, while accommodating user-defined risk factors.

The particle swarm optimization (PSO) algorithm^28^is another robust algorithm for global optimization employed in well placement problems^29,30^. Humphries and Haynes^31^employed particle swarm optimization alongside mesh adaptive direct search to tackle the joint optimization of well placement and well control parameters. The research compares simultaneous and sequential approaches, revealing that focusing on positional parameters within a staged optimization framework can yield more effective solutions compared to a fully simultaneous approach. Particle Swarm Optimization has been applied by Khan and Awotunde^32^as an optimization algorithm to determine the optimal parameters in field development planning, including well types, locations, and production rates. PSO was integrated into the generalized field development optimization procedure effectively, offering a robust alternative to traditional optimization methods. In Onwunalu’s^3^study, Particle Swarm Optimization was developed and applied to optimize the type and location of new wells in oil field development. A new procedure was introduced to handle large-scale field development, optimizing well patterns and their geometric configurations while accounting for local reservoir variations. Additionally, a meta optimization process was implemented to optimize PSO algorithm parameters, improving its performance in well placement tasks across various benchmark and realistic scenarios. Biswas^33^ developed a novel hybrid optimization approach that combines cellular automata with grey wolf optimization and particle swarm optimization to tackle the nonlinear and constrained mathematical optimization problem of wellbore trajectory design. They demonstrated improved performance in terms of Pareto optimality and solution diversity, achieving significant reductions in the Inverted Generational Distance (IGD) compared to standard methods, along with optimal values for spacing and spread metrics.

Additional studies have been conducted that combine GA with PSO. Sheikhalishahi^34^combines GA and PSO by using GA to generate a population of solution pairs (n, r) and then applying PSO to refine the r vector in a selected subset of these pairs. GA handles the broad exploration of the solution space, while PSO focuses on improving specific solutions within that space. Garg^35^operated hybrid method by initializing a swarm of particles and their velocities, updating their positions based on both personal and global experiences, and periodically applying GA to selected particles to refine the search for optimal solutions. Roy and Das’s^36^ introduced a fusion factor, which determines the proportion of particles updated using PSO versus GA, balancing exploitation and exploration by varying between 0 and 1. In practice, this involves generating an initial population, evaluating fitness, and then applying PSO and GA operations to subsets of particles based on the fusion factor to iteratively improve the solution.

The framework of this study is made up of three main parts: the Eclipse reservoir simulator, some optimization techniques especially a developed algorithm (GA-PSO), and a Net present value calculation module as the target function. The novel integrated optimization algorithm (GA-PSO) specifically designed to enhance well placement strategies by maximizing net present value. By combining the strengths of genetic algorithms and particle swarm optimization, this approach ensures a more effective exploration of the search space and avoids local optima. The algorithm’s unique ability to retain and reintroduce less qualified individuals for further optimization sets it apart from traditional methods. This work addresses critical parameters such as different scenarios of well arrangements in different patterns or even scatter manner of whether vertical or horizontal wells, well direction, displacement, and length, offering a more innovative and cost-efficient solution to well-placement challenges. In this structure, the well locations are optimized if the maximum net present value is obtained as our target function.

## Theory

### Genetic algorithm

GA functions as a computational emulation of natural selection’s evolutionary process, where solutions compete for survival. In GA, possible solutions to the optimization problem are represented as individual entities within a population. The quality of a solution, known as its fitness, progresses as the algorithm advances through iterations, moving from one generation to the next. During simple GA, in each generation, individuals are sorted from the best to the worst according to their fitness.

To generate new generations from existing ones, GA employs three operators, selection (Immediate transfer of the highest-performing individuals, chosen according to their fitness levels), crossover (two individuals are combined to create two new offspring, or random parameters from one individual are selected to swap with random parameters from another individual) and mutation (a random individual is selected to changing some of its parameters)^37,38^. Upon the conclusion of the simulation, the top-performing individual (possessing the highest fitness) embodies the solution to the optimization challenge. The GA performance has been expressed meticulously elsewhere^39,40^.

### Particle swarm optimization (PSO)

Like GA, the PSO algorithm has various operators to create new solutions from the existing ones and employs random elements to avoid solutions getting stuck in local optima. The genetic algorithm (GA) and the particle swarm optimization (PSO) algorithm have different approaches. The GA can select the best option among many applicable strategies in the production of new generations^41,42^depending on specific optimization problems^37,43^, while the PSO algorithm has a different structure with only one main operator, known as the “velocity” equation.

The velocity equation comprises multiple elements that propel the particle through the search space with a specific speed. In PSO, each particle retains a memory in addition to random initial velocity. This velocity dictates individual particle’s search direction and is recalculated during every cycle of the algorithm^28^.

The GA and PSO algorithms diverge in the quantity of vectors linked to each individual or particle. In GA, every individual has one solution vector, while in PSO, each particle is linked to three vectors: its present location, velocity, and historical best position.

The PSO algorithm employs a collaborative search approach for optimization at which the particles are allowed to interact with one another. This interaction facilitated through neighborhoods, where a particle can solely engage with others within its designated neighborhood^44^. The global best (Gbest) and local best (Lbest) PSO variants are determined according to the number of used neighborhoods^45^. In Gbest PSO, a singular neighborhood encompassing all particles is utilized while in Lbest PSO, multiple neighborhoods are utilized, allowing particles to belong to more than one neighborhood for optimization purposes.

### Integrated optimization Algorithm

Another optimization technique that has been employed in this study is the integrated optimization algorithm (GA-PSO). In this algorithm, first, a random initial population (algorithm’s individuals can be chromosomes as in the genetic algorithm or particles in the PSO algorithm) is created in the searching space at which the competency of each of its individuals is determined by a fitness function (here NPV).

After sorting the particles by algorithm, depending on the crossover percentage some of the better individuals are chosen, and new chromosomes are generated. The operator is structured to enable effective information exchange and inheritance between generations. At the next step, the mutation operator is applied guarantee the introduction of fresh genetic material into the chromosome to promote their performance. This operator also enables exploration of various regions within the search space and prevents the algorithm from trapping in local optimums.

**Fig. 1:**
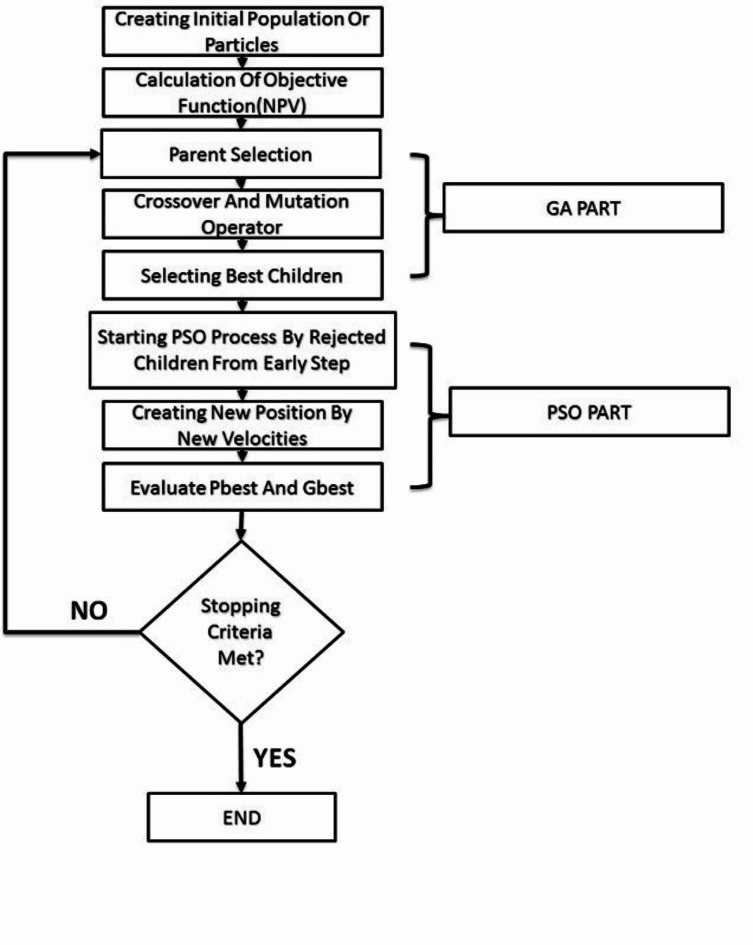
Flowchart for Integrated Algorithm(GA-PSO).

New offspring that were generated from crossover and mutation operators are sorted according to their fitness, and better children are chosen based on population size. The others who didn’t get enough qualifications to continue their life from early steps are used by a PSO algorithm to obtain new velocities and situations. This means the memory characteristics of the PSO algorithm let unqualified individuals return to the process by means of new velocities. In fact, this algorithm prevents unqualified individuals from being omitted and gives them a new chance to get great potential for finding the best solutions. The process is stopped when the specified convergence criterion is satisfied then the results will be analyzed. The flowchart of the integrated optimization algorithm is shown in Fig. 1.

The distinction between the proposed GA-PSO approach and previous studies^34–36^ is rooted in the integration and application of Particle Swarm Optimization (PSO) and Genetic Algorithm (GA). Unlike other studies that utilize a hybrid method to concurrently leverage the strengths of both algorithms, our approach runs GA and PSO sequentially. This strategy allows unfit individuals generated by the GA to receive a second opportunity through PSO, where the most effective candidates are identified and reintegrated into the optimization process. Allowing unqualified individuals to re-enter the GA-PSO process with new velocities enhances both search efficiency and solution quality. It promotes exploration, maintains diversity, and can help in discovering and refining higher quality solutions while reducing the risk of getting stuck in local optima.

## Model description

### Reservoir model description

The reservoir model in this study is a square synthetic reservoir that extends over a 40,000 by 40,000 square feet area and is presently being assessed for full development and all wells were completed at a depth of 8000 feet. The model is considered homogeneously in that its porosity is 17% and permeability in x, y, and z directions are 100, 100, and 10 md, respectively. The model employs an isotropic permeability, with a vertical-to-horizontal permeability ratio of 0.1. Initial reservoir pressure is 4500 Psi and production is on pressure control at 2000 psi. It should be mentioned that every case was simulated for 20 years.

### Model variables and scenarios

In this study, the utilization of the PSO, GA, and integrated algorithm (GA-PSO) to optimize the initial number of wells, location of existing and new wells in well pattern and type of them have been described. In order to maximize NPV as the objective function, a variety of optimization problems such as small displacement of existing wells, well direction, length of horizontal wells and number of them have been considered as input parameters.

### Economic model

Generally, the purpose of well placement optimization is to improve profit and decrease costs. The type of objective function that must be optimized is one of the vital input parameters that should be defined in an optimization problem. Two types of objective function known as the traditional objective function, can be defined in well-placement optimization problems: cumulative production and NPV^46^. After the simulation of each individual, objective functions can be calculated using the simulation output file. While cumulative oil production provides a singular metric, the total oil volume at the end of the simulation (NPV) considers the project’s economic aspects more comprehensively.

The employed economic model in this study is based on Yeten’s study^18^ and is determined by a fixed yearly effective discount rate according to the following Eq. (1):

1 $$NPV=\sum\limits_{n=1}^{y}\sum\limits_{P=o,g,w}(\frac{1}{(1+i)^n}\times Q_{P}^{n}\times C_P)-C_{capex}$$

Where Q_p_^n^ represents the phase p production rate during the n^th^ year, C_p_ is the costs that associated with phase p, i denotes the annual percentage rate (APR), Y is the total number of discount years and C_capex_ is drilling and completion cost. C_capex_ can be calculated by the Eq. (2):

2 $$C_{capex}=\sum_{i=1}^{well \,count}(C_d+L_{tot, i}\times C_{drill})$$

Where C_d_ signifies the capital cost per well, inclusive of platform expenses and drilling costs, C_drill_ denotes the unit drilling cost per foot, and L_tot, i_ is the total length of ith well. The values utilized in this study are presented in Table 1, derived from one of the current ongoing drilling projects.

**Table 1:** Utilized values for the economic model calculations.

Parameters	Values
Discount rate (%)	10
Oil price ($/bbl)	80
Desalination cost ($/bbl)	2
Drilling cost ($/m)	2000
Fixed costs of Well (MM$/Well)	20

## Results and discussion

### Sensitivity analysis

Following sensitivity tests on the integrated algorithm (GA-PSO), optimal parameters were chosen, leading to a more detailed analysis aiming to establish an optimal field development strategy. The sensitivity analysis for both algorithms and scenario parameters has been done in the next sections.

#### Algorithm parameters

In this part, the effect of different algorithm parameters such as percentage of crossover and mutation operators, number of particles or population size, and number of each of GA and PSO individuals that were used in the integrated algorithm (GA-PSO) are compared. Knowing the best values of these parameters could improve algorithm performance in the search process.

**Fig. 2:**
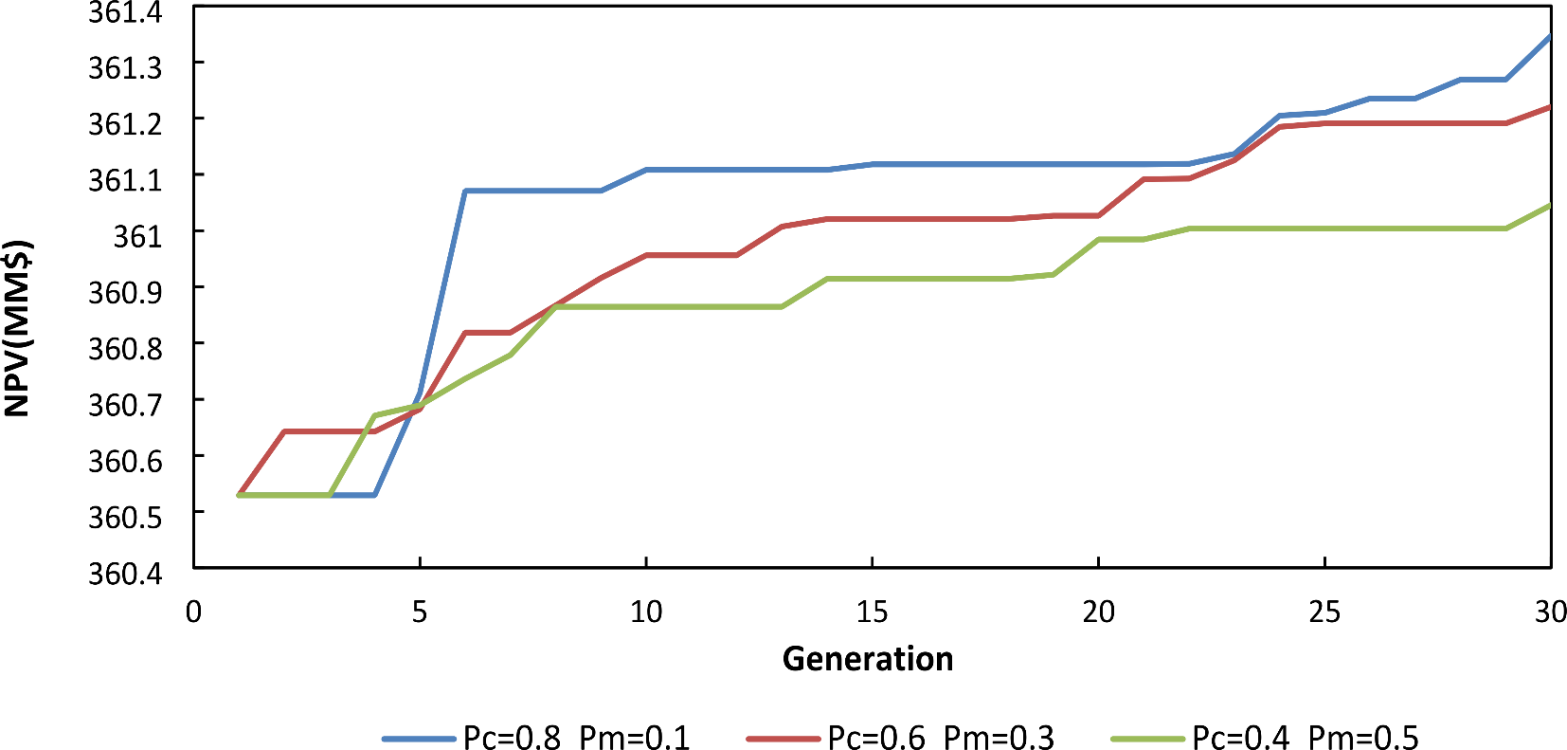
NPV for various mutations and crossover percentage.

As shown in Table 2; Fig. 2, when sensitivity tests have been performed on crossover and mutation percentages holding other parameters constant, the results show better performance in lower value of the fraction of mutation operation (Pm = 10%) and higher value of the fraction of crossover operation (Pm = 80%). It should be mentioned that to ensure the reliability of the results, each curve in Fig. 2 is an average of three runs with the same initial population values.

**Table 2:** Variation in mutation and crossover percentage.

	Generation number	Population size	Crossover percentage	Mutation percentage	Average NPV(MM$)
**Case1**	30	20	0.8	0.1	361.3466
**Case 2**	30	20	0.6	0.3	361.2202
**Case 3**	30	20	0.4	0.5	361.0454

**Fig. 3:**
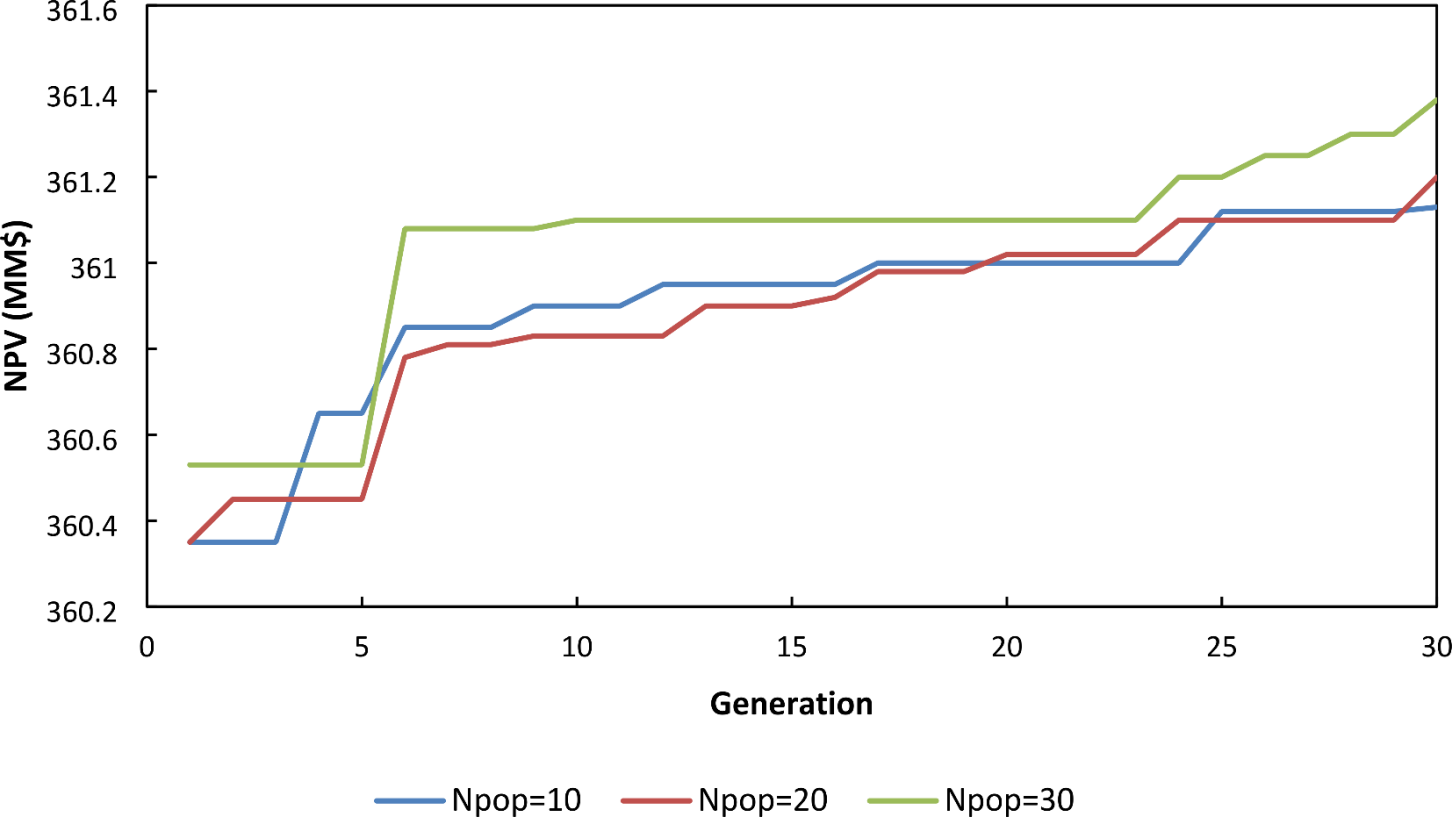
NPV for different population sizes.

In the next step, the population size varied to 10 and 30 while other parameters were kept constant in their previous calculated optimums and compared with the results of a population size of 20. As illustrated in Fig. 3, the results showed that the higher the population size, the better the answers. It can be attributed to the greater number of function evaluations.

**Fig. 4:**
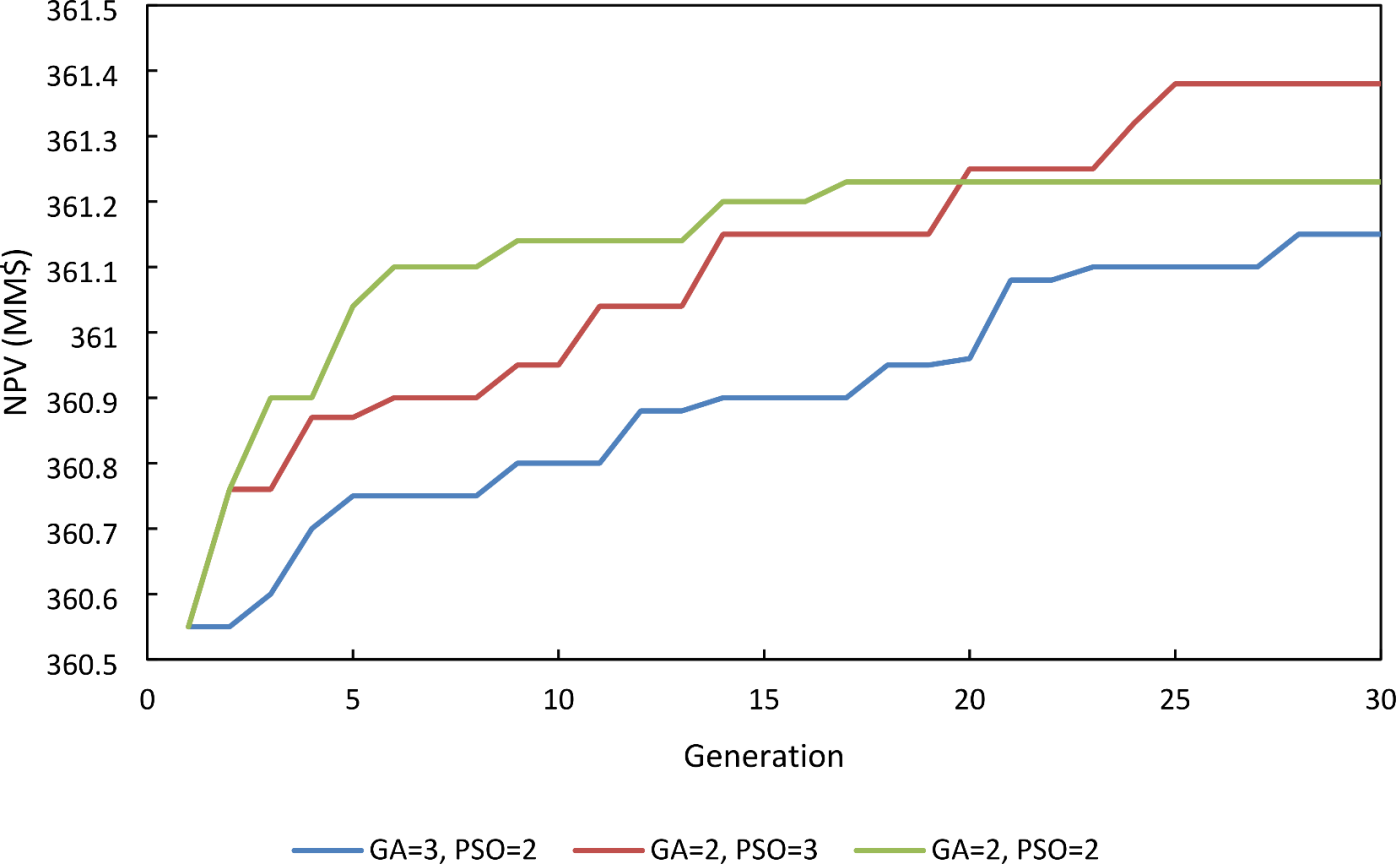
NPV for different numbers of sub-algorithms.

To determine the effect of the proportion of the numbers of different main sub-algorithms (GA and PSO) used in the integrated algorithm (GA-PSO), different values of the number of PSO and GA sub-algorithms in the integrated algorithm (2PSO-2GA, 2PSO-3GA, and 3PSO-2GA) were examined. As illustrated in Fig. 4, the result shows that the best performance was obtained at an equal number of sub-algorithms while the number of function evaluations was higher in the other two proportions.

According to the performed sensitivity analysis on the integrated algorithm (GA-PSO), optimum results were obtained at a crossover percentage of 80%, mutation percentage of 10%, population size of 20, Generation number of 50, and sub-algorithm proportion of 1PSO-1GA.

#### Number of wells

Before optimizing the wells’ locations and other operational parameters affecting well pattern optimization, a quick screening test to evaluate the optimum number of wells (that is one of the algorithm’s variables in every scenario) was performed. To this end, various numbers of wells of 6, 8, 10, 12, 13, 14, 15, 16, and 18 in a disordered manner have been considered in the optimization problem separately.

**Fig. 5:**
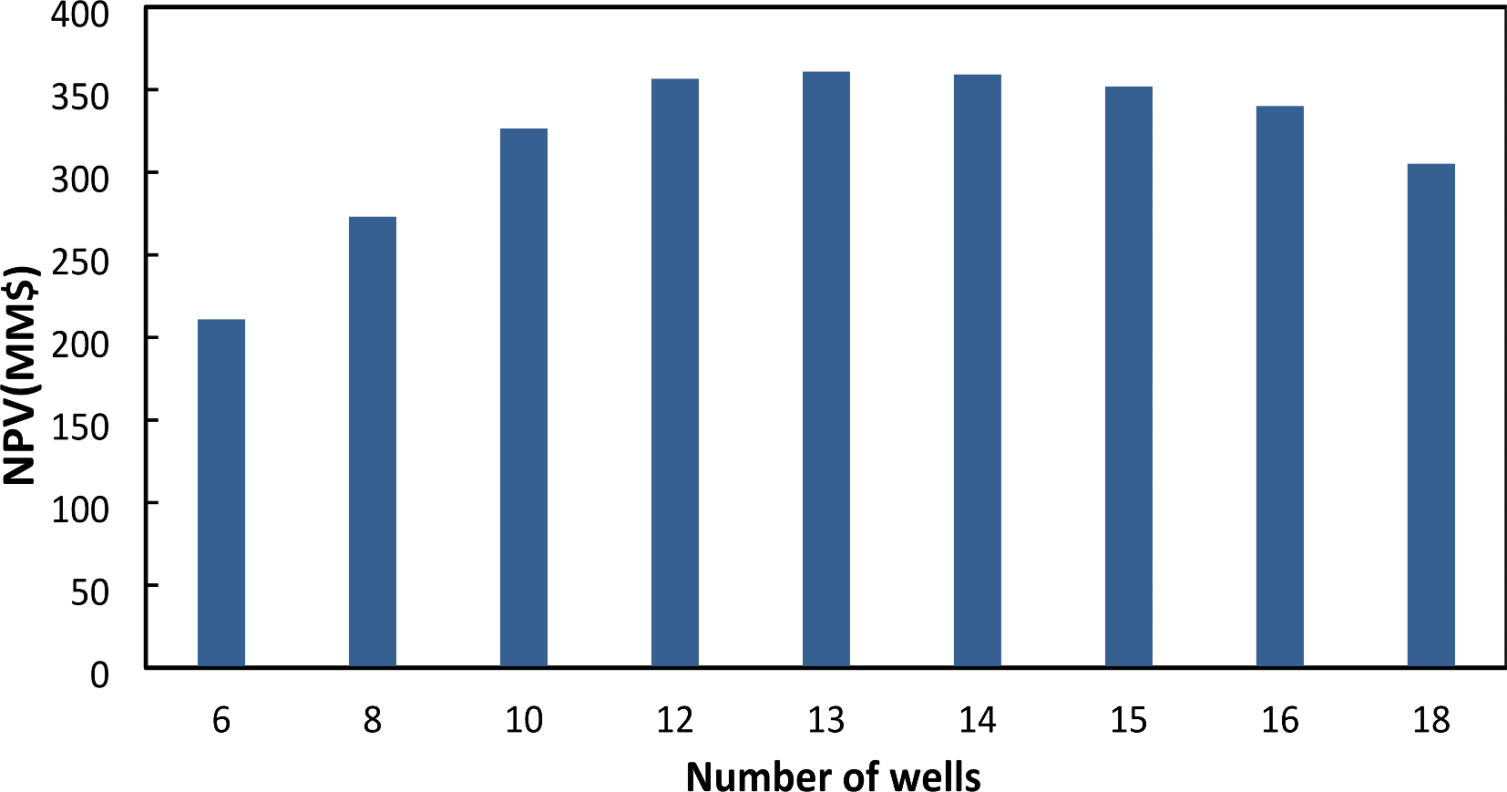
NPV vs. Number of Wells.

As shown in Table 3; Fig. 5, NPV increases as the number of wells increases from 6 to 13 wells and decreases from 13 to 18 wells. Therefore, 13 wells have been considered as the optimum number of wells in the evaluation of integrated algorithms performance and its speed of convergence.

**Ta﻿bl﻿e 3:** NPV and related number of wells in optimum locations.

Number of wells	NPV
6	210.9457
8	273.1554
10	326.5885
12	356.6176
13	360.9351
14	359.2025
15	351.9371
16	340.0462
18	305.1941

### Optimizing scenarios

After optimization of the algorithm parameters and the number of wells, the integrated algorithm has been employed to optimize well placement in a synthetic reservoir model.

#### Well pattern optimization (location, type of well, spacing)

The type, number, location, and pattern of wells and the distance between them are some of the most challenging problems in oil and gas field development. In order to find the best configuration of these variables using the developed algorithm and compare its performance with other ordinary algorithms, an optimizing code has been provided. To achieve this purpose according to the aforementioned reservoir size, location variables and other parameters are generated as random numbers by the algorithm. Then the created numbers get their real values using the maximum and minimum dimensions that we set as input parameters.

At first, in order to determine the optimum well locations, the number of injection and production wells, and which of them must be injection wells, random sets of combinations have been generated using the mathematical concept of combination law^47^, which is a way of selecting items from a collection. This approach allowed us to systematically select subsets of possible well locations from a larger set facilitating a comprehensive exploration of potential scenarios. By generating multiple initial configurations through a series of runs, we ensured that the same optimal configurations were consistently achieved. When the data has been set in the data file of the reservoir simulator, the simulator runs the model, then the production data has been exported as an output data file for NPV evaluation as the objective function.

**Fig. 6:**
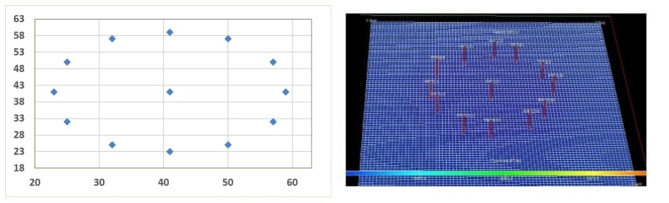
The placement of 13 production wells with zero injection wells as the optimum case.

All three GA, PSO, and GA-PSO algorithms suggest 13 producing wells and zero injection wells as the optimum number of wells. Furthermore, all of them reported the same location at the end of the optimization process. The best configuration was at 41 grids in the x direction and 41 grids in the y direction. At the optimized configuration, all wells had a distance of 9.5 grid cells from their adjacent wells and 18 grid cells from the center well. Figure 6 illustrates the ultimate optimal outcomes regarding the positioning of 13 production wells.

**Fig. 7:**
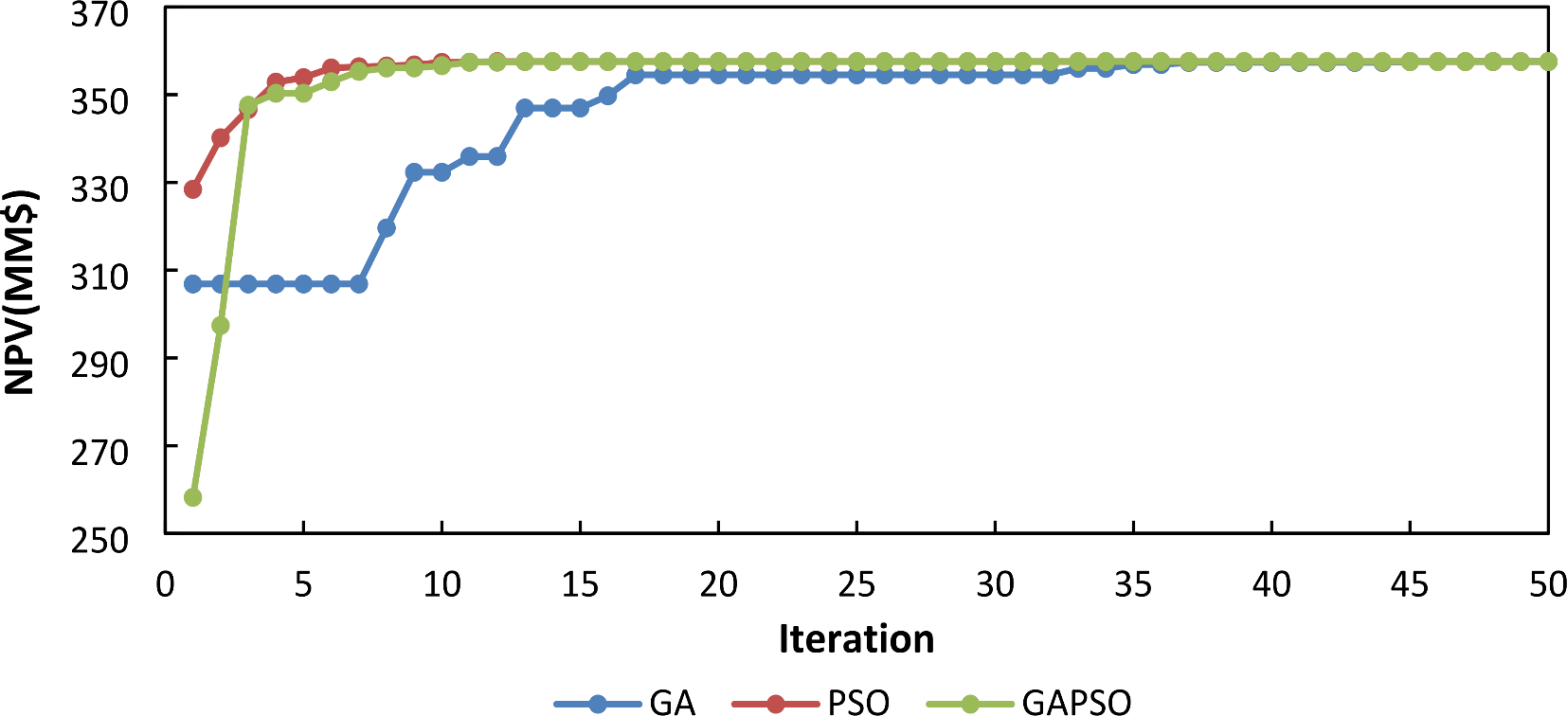
Well pattern optimization using Algorithms.

The convergence speed is an important parameter that makes distinctions among different algorithms. The core challenge in optimizing lies in the significant computational burden posed by the reservoir simulation, rather than the steps of optimization algorithm. Therefore, choosing an algorithm to reduce the number of required simulations, thereby enhancing overall efficiency and effectiveness in achieving optimal solution is the main concern. The speed of convergence, defined by the slope of the NPV versus iteration curve, is a critical factor in evaluating algorithm performance. The GA-PSO method demonstrates a significantly improved convergence rate in first iterations, highlighting its effectiveness in reaching optimal NPV values. The reliability of the results was rigorously verified through multiple iterations, each involving the random generation of initial values, consistently yielding the same outcomes. To provide deeper insight into the speed of convergence, one of these results is illustrated in Fig. 7.

**Fig. 8:**
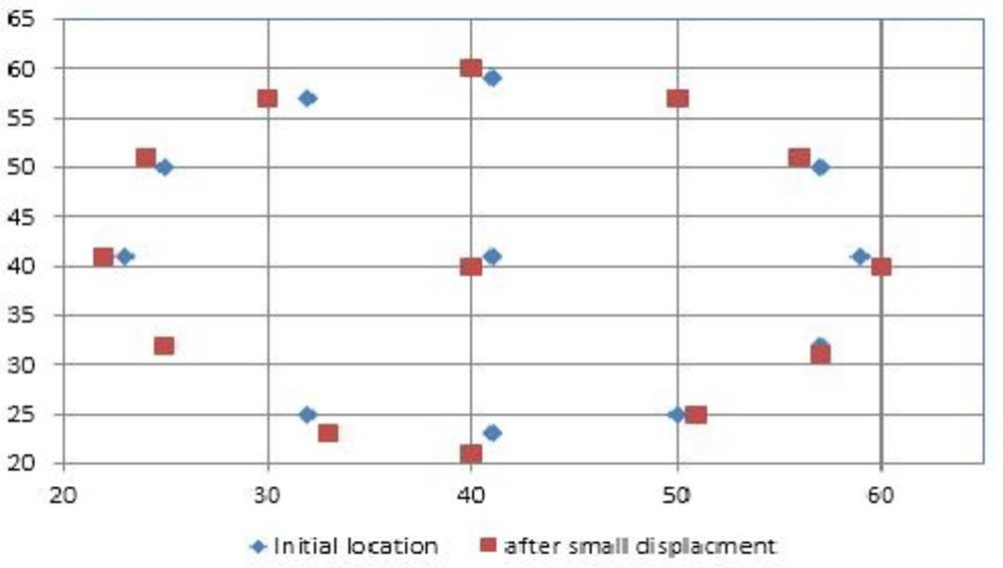
Schematic view of the location of wells before and after a small displacement.

**Fig. 9:**
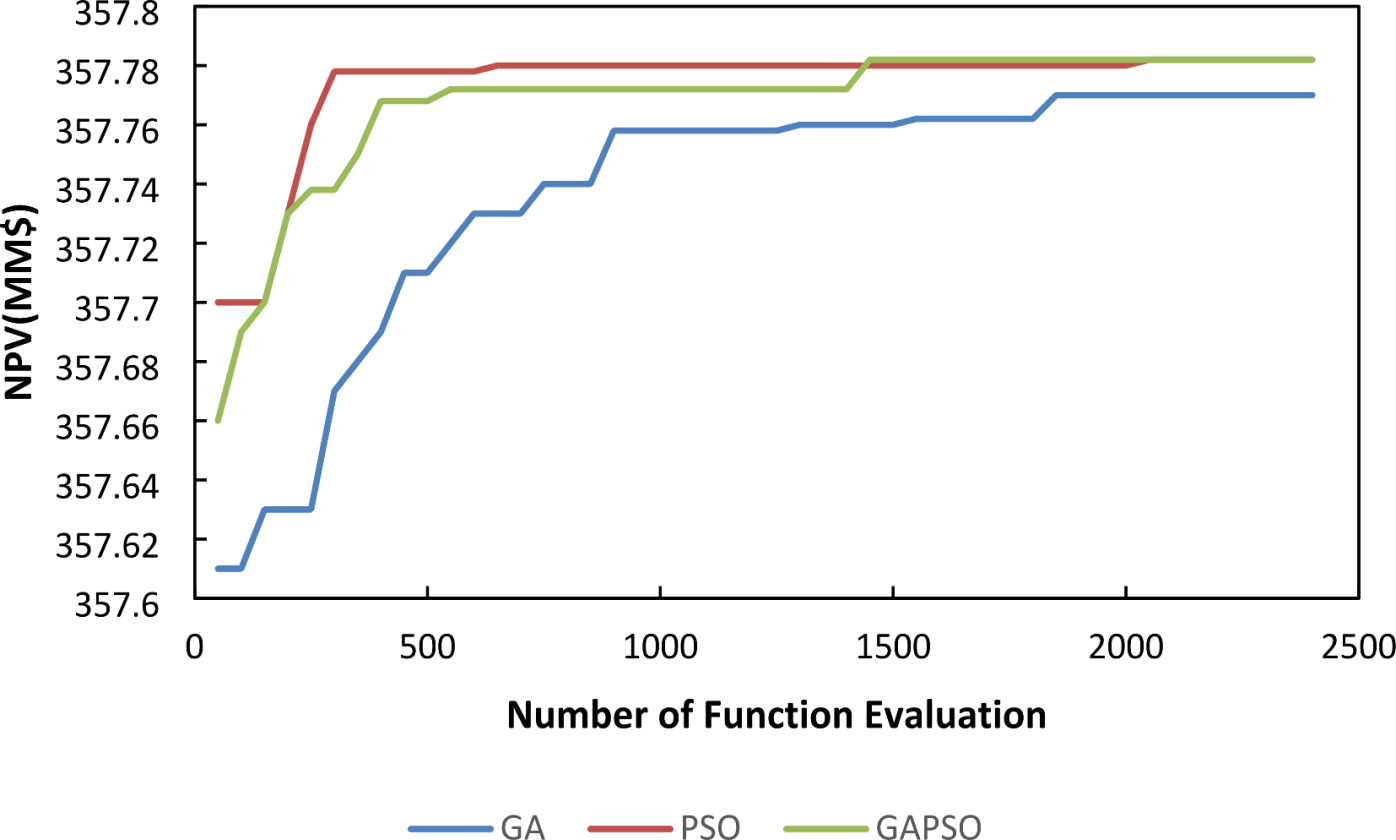
The trend of change in NPV in case of small well displacement.

In order to ensure the optimum well locations, the wells were allowed to be placed near the original location without disarranging the well pattern. So, the number of the generated variable by the algorithm is twice the number of initial wells, and the locations were changed between a minimum and maximum margin in both x and y directions that have been set previously as input parameters. A penalty function was considered to prevent placing two wells near the minimum spacing. Figure 8 is a schematic view of old and new places provided by the simulator. Figure 9 compares the performance of each algorithm showing that after performing the optimization process, a 0.1% increment in NPV was reported.

#### Infill placement optimization

Developing a giant field in order to achieve maximum ultimate oil recovery (UOR) through optimal placement of new production and injection wells is a challenging task. Additionally, converting existing production wells into injection wells and vice versa represents other pragmatic methods to enhance the recovery.

Although lots of methods are recommended in field development, none offer a comprehensive solution. In field development, firstly the new well’s location will be determined, and then its impact on the ultimate oil recovery will be investigated. Since most of the time during this process we have to handle a substantial number of existing wells and evaluate numerous potential infill options, designing an infill drilling program becomes a complex task that makes it a time-consuming and expensive process. It is crucial to schedule the drilling of these wells at suitable intervals and locations to prevent or minimize interference with their drainage patterns.

In this study, in order to establish guidelines for drilling new production wells or injectors an integrated approach has been employed. This method presents a competitive alternative with EOR processes in terms of the recovery factor requiring significantly lower investments and operational expenses.

**Fig. 10:**
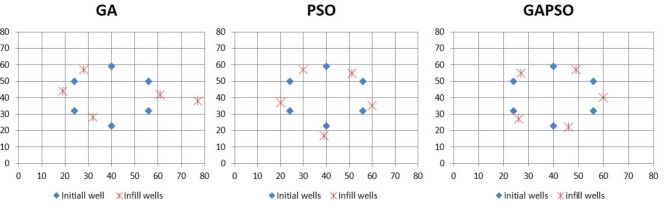
Schematic location of infill wells in different algorithms.

In order to perform the infill placement optimization, the optimum placement of wells from the previous section with the assumption that just 6 of them have been completed in the early three years, have been employed. So, an algorithm is performed to find the best number of infill wells, their types, and locations that will be placed after three years, for the next 20 years simulation period. As shown in Fig. 10, all the algorithms found 5 wells as the best number of infill wells and all of them are production wells. The best method among the three examined algorithms was determined based on the convergence speed to the best optimum location that leads to the highest NPV.

**Fig. 11:**
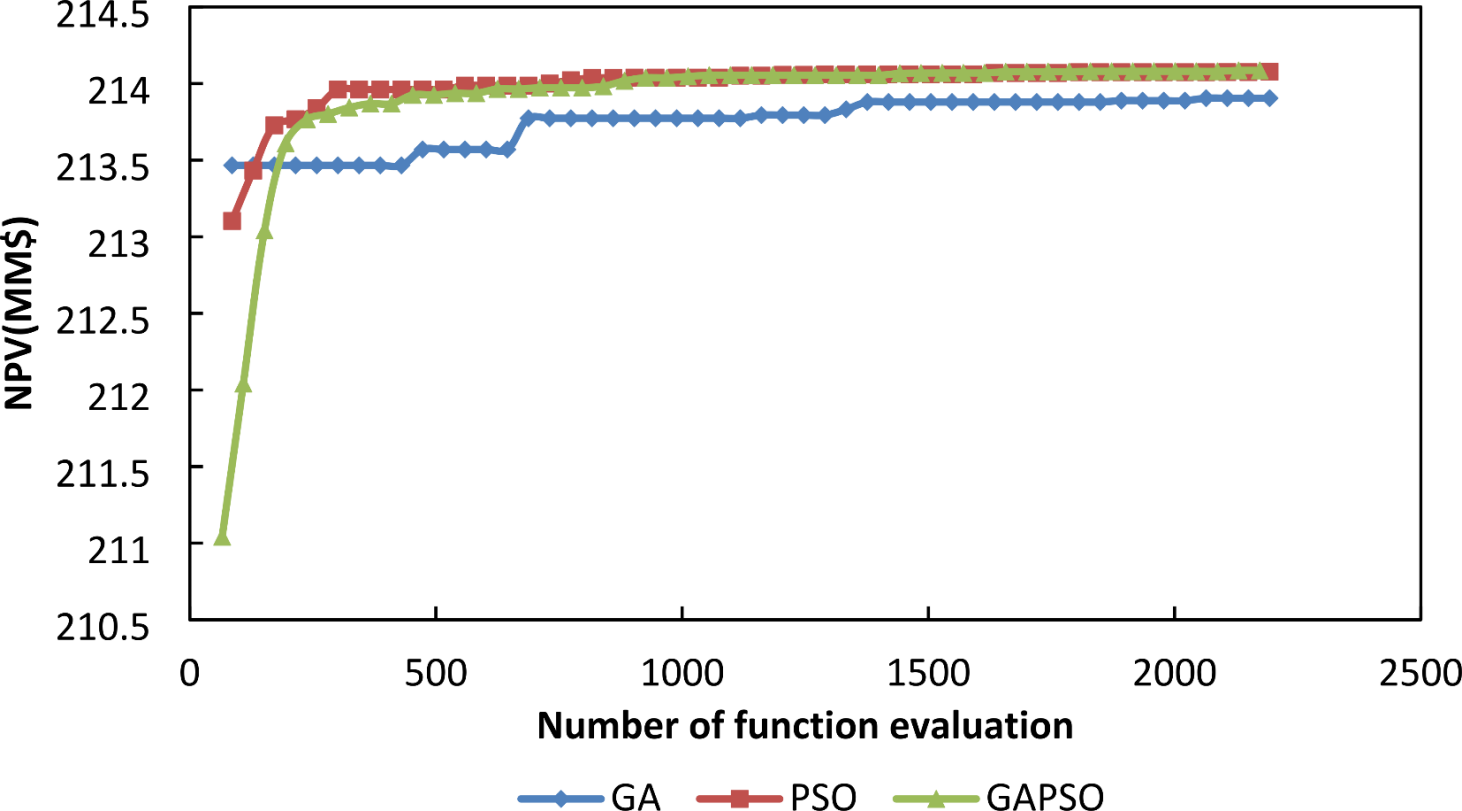
NPV Vs. Number Of Function Evaluation For Different Algorithm In Infill Placement Optimization.

Figure 11 shows the NPV variation based on the number of considered function evaluations in different algorithms during the infill placement optimization. According to this figure, although the initial random population estimated by the Integrated Algorithm is the worst among the three algorithms, it has the best convergence speed. Interestingly, the location of new wells to achieve the best NPV is located in places that fill the vacant space of pattern in the reservoir model and the Integrated algorithm is faster than the other two examined algorithms in finding these places.

#### Horizontal wells optimization

Horizontal wells due to their crucial role in increasing efficiency and reducing costs have received substantial attention. In this study, the optimization of horizontal wells as an effective technique in field development has been investigated. To achieve this goal, first of all, the optimum number of horizontal wells by the Combination concept of “r” objects (the number of horizontal wells) of “n” objects (the total number of wells) is determined, and then the places of horizontal wells are chosen. After determining the length and direction of drilling and performing the simulation, considering the drilling cost of the horizontal wells per foot, the output data from simulators are employed in economic evaluations. In our optimization algorithm, each potential horizontal well length is evaluated using the objective function presented in Eq. (1) that incorporates production rates, drilling costs, and overall economic viability, ensuring that the algorithm effectively balances the economic outcomes for both very long and very short wells. It should be noted that special penalty functions have been employed to avoid the possibility of horizontal wells overlapping by imposing a 5% reduction in Net Present Value (NPV) for each step where the distance between wells falls below five grid blocks. This distance-based mechanism assumes that closer wells will interfere with each other’s drainage areas, leading to reduced oil production.

**Fig. 12:**
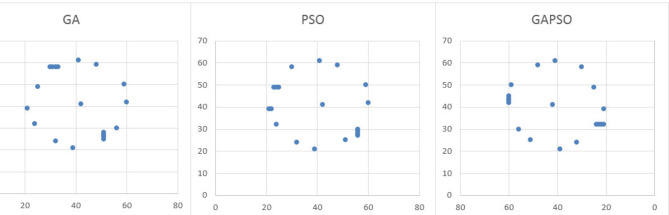
Schematic view of the optimum case of horizontal drilling for different algorithms.

After the implementation of different algorithms on the optimized well pattern, it has been observed that the horizontal wells tend to be completed toward the outside of the well pattern. It should be mentioned that in most cases, the horizontal wells were in the farthest position relative to each other. While PSO reported 3 horizontal wells as an appropriate number of wells to be drilled horizontally, the developed method (GA-PSO) and GA reported 2 wells as an optimum number of horizontal wells. The details of the optimized location of wells can be seen in Fig. 12. The differing recommendations from the GA and GA-PSO algorithm (two horizontal wells) and the PSO algorithm (three horizontal wells) reflect the possibility of having different approaches to the optimization process in reaching the final objective function, even when the end results exhibit relatively close values, allowing for a comprehensive analysis that favored the more economically efficient recommendation.

**Fig. 13:**
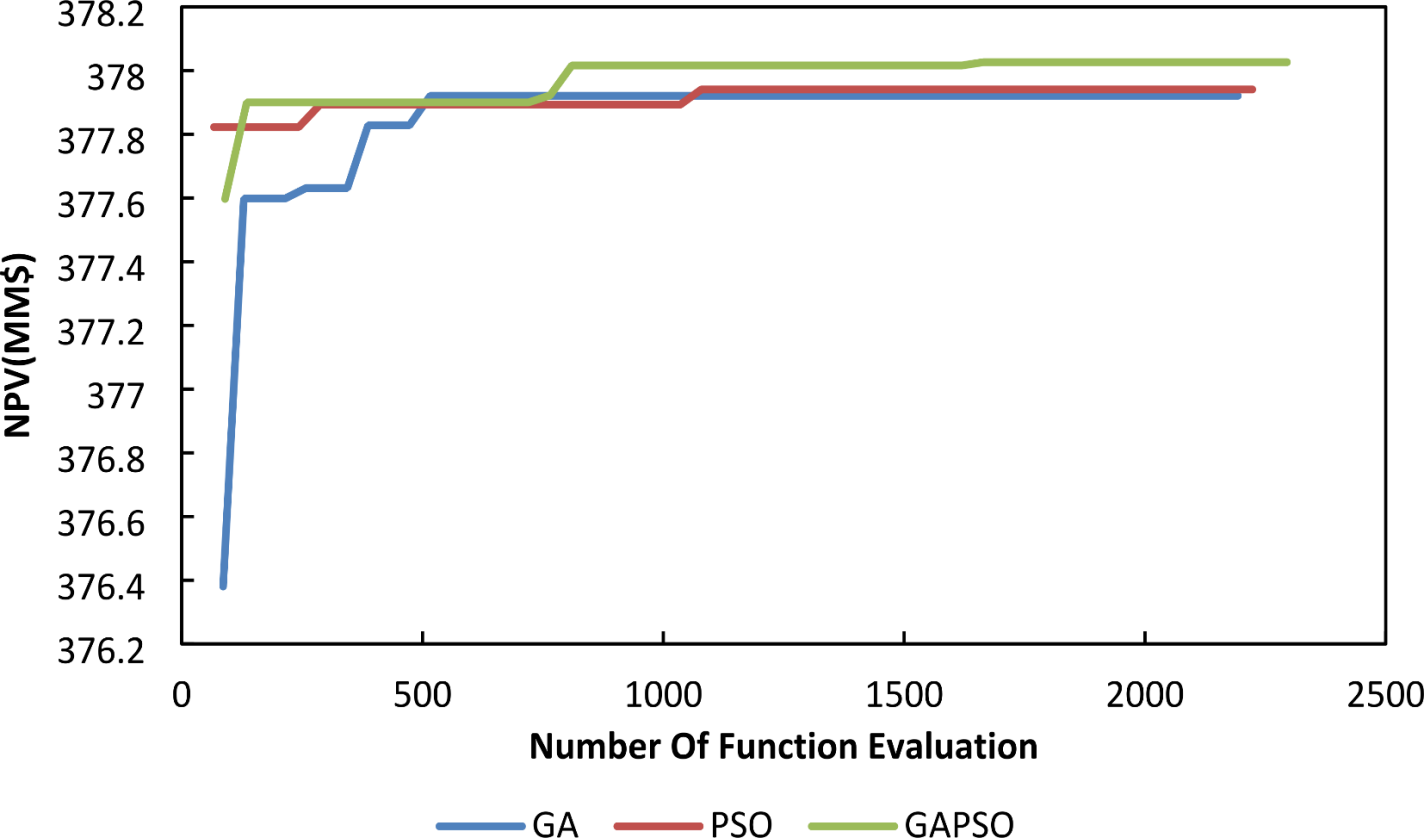
NPV vs. Number of function evaluations for horizontal drilling in different algorithms.

Having a glimpse at Fig. 13 which shows the trend of NPV vs. the number of simulations, it can be found that the proposed solution by the integrated algorithm (GA-PSO) leads to more profits than the other two algorithms and also the best and optimum number of horizontal wells is 2 wells and are in farthest distance from each other. It is interesting to know that performing horizontal drilling operations increased the current NPV by 6.25% of the earlier amount, which is a significant value in field scale.

### Performance of optimization algorithms

To quantify the performance of the implemented algorithms, two metrics were utilized: Generational Distance (GD) and Convergence Rate. The GD performance indicator quantifies the distance between solutions and the reference points^48^. Let’s define the points produced by the algorithm as the objective vector set A = {a_1_, a_2_, …, a_n_} and the reference points as Z = {z_1_, z_2_, …, z_n_}. In this study, the reference points were set to the same value, corresponding to the maximum possible NPV achieved. The Generational Distance is calculated as follows^48^

3 $$\:GD=\:\frac{1}{n}\:\sqrt{\sum\:_{i=1}^{n}{d}_{i}^{2}}$$

Here, d_i_ denotes the Euclidean distance from a_i_ to its nearest reference point in Z. This effectively represents the average distance from each point in A to the closest point on the NPV. A smaller Generational Distance indicates better performance in achieving the maximum possible NPV.

Convergence Rate measures how rapidly the algorithms converge to a target value over successive iterations^49^. The rate of convergence is defined in terms of the difference between iterations of the algorithm. For an objective function f(x) with optimal value of f^*^, the convergence rate can be calculated as

4 $$\:\text{C}\text{o}\text{n}\text{v}\text{e}\text{r}\text{g}\text{e}\text{n}\text{c}\text{e}\:\text{R}\text{a}\text{t}\text{e}=\:\frac{\:\:{f}^{*}-\:f\left({I}_{t}\right)}{\:{I}^{*}-\:{I}_{t}}\:$$

Here, I_t_ represents the iteration number, and I^*^ denotes the iteration at which the optimum value is achieved. To obtain a single convergence rate for each algorithm, the mean of the convergence rates across all iterations is reported by comparing the first iteration with I^*^.

The results of these two metrics for various simulations are presented in Table 4. Cases in which the optimum value is not achieved are denoted with a dash symbol. As observed, GA-PSO exhibits the highest convergence rates among the algorithms, indicating superior performance in achieving maximum NPV with fewer iterations compared to the other two algorithms. The GA fails to reach the maximum NPV in three out of four cases, while the PSO demonstrates an acceptable convergence rate but falls short of achieving the desired NPV in one case.

Additionally, for the Generational Distance metric, results that are closer to the final NPV (i.e., lower values) indicate better performance. In this metric, both PSO and GA-PSO demonstrate acceptable performance, with PSO exhibiting superior results. Considering the significance of the convergence rate in achieving the maximum NPV more quickly with fewer time-consuming simulations, along with the importance of reaching the maximum NPV in all cases with GA-PSO, this algorithm can be regarded as the preferred choice.

Finally, it is worth mentioning that the significant difference between the reported values for the well pattern case compared to the other cases in Table 4 can be attributed to the fewer number of iterations in this scenario and the potential for drilling new wells, which allows for meaningful changes in the NPV.

**Table 4:** Performance metrics of optimization algorithms across various simulations.

	Generational distance			Convergence rate			
	GA	PSO	GAPSO	GA	PSO	GAPSO	
Well pattern	4.3	1.2	2.3	1.4	2.9	9.9	
Small well displacement	12.2e-3	4.7e-3	5.9e-3	-	4.1e-5	6.6e-5	
Infill placement	9.9e-2	3.1e-2	4.5e-2	-	1.2e-3	3.6e-3	
Horizontal drilling	2.7e-2	1.6e-2	1.4e-2	-	-	6.5e-5	

## Conclusions

Key insights and reflections from this paper are as follows:

1. This study presents an innovative approach to optimizing well placement and operational settings in reservoir development by introducing an integrated GA-PSO algorithm. The combination of genetic algorithms (GA) and particle swarm optimization (PSO) harnesses the strengths of both methods, resulting in enhanced performance over traditional standalone algorithms.
2. The integrated GA-PSO algorithm demonstrated its superiority in maximizing Net Present Value (NPV), consistently outperforming GA and PSO in identifying optimal well placements. Notably, the algorithm’s ability to converge more quickly to the optimal solution reduced computational time, making it a more efficient tool for reservoir engineers.
3. In addition to performance improvements, the integrated algorithm excelled in avoiding local optima, a common challenge in optimization problems. This novel capability allows for a more thorough exploration of the search space, leading to better outcomes in well placement and operational settings. The study’s sensitivity analysis further emphasized the importance of fine-tuning algorithm parameters, highlighting how adjustments in crossover and mutation rates can significantly impact optimization success.
4. The work also offers practical implications for real-world applications. While the study was conducted using a synthetic reservoir model, the developed GA-PSO algorithm can be readily adapted to actual field scenarios. This adaptability underscores its potential as a valuable tool for optimizing well placement in diverse reservoir conditions, ultimately enhancing oil recovery and economic returns.
5. Further research could also focus on refining the integrated algorithm, perhaps by incorporating other optimization techniques or adjusting parameters based on specific reservoir characteristics. Testing the algorithm on a variety of reservoir types, including heterogeneous and anisotropic reservoirs, could provide insights into its generalizability and robustness. The broader economic and environmental impacts of using such advanced optimization algorithms in oil and gas field development could also be explored.

## Data Availability

All data generated or analysis during this study is available from the corresponding author base on a reasonable request.
